# Dissociation between memory retention across a delay and pattern separation following medial prefrontal cortex lesions in the touchscreen TUNL task^☆^

**DOI:** 10.1016/j.nlm.2013.01.010

**Published:** 2013-03

**Authors:** Kathryn A.L. McAllister, Lisa M. Saksida, Timothy J. Bussey

**Affiliations:** a University of Cambridge Department of Psychology, Downing Street, Cambridge, CB2 3EB, UK; b Wellcome Trust and MRC Behavioural and Clinical Neuroscience Institute, University of Cambridge, Cambridge CB2 3EB, UK

**Keywords:** Prefrontal cortex, Working memory, Pattern separation, Touchscreen, TUNL, Nonmatch-to-position

## Abstract

The neural structures that support the retention of memories over time has been a subject of intense research in cognitive neuroscience. However, recently much attention has turned to pattern separation, the putative process by which memories are stored as unique representations that are resistant to confusion. It remains unclear, however, to what extent these two processes can be neurally dissociated. The trial-unique delayed nonmatching-to-location (TUNL) task was developed to assess spatial working memory and pattern separation function using trial-unique locations on a touch-sensitive screen (Talpos, McTighe, Dias, Saksida, & Bussey, 2010). Using this task, Talpos et al. (2010) showed that lesions of the hippocampus led to both impairments with a 6 s delay, and impairments in pattern separation. The present study shows that lesions of the medial prefrontal cortex lead to a different pattern of effects: impairment at the same, 6 s delay, but no hint of impairment in pattern separation. In addition, rats with medial prefrontal lesions were more susceptible to interference in this task. When compared with previously published results, these data show that whereas the prefrontal cortex and hippocampus likely interact in the service of working memory across a delay, only the hippocampus and not the medial prefrontal cortex is essential for pattern separation.

## Highlights

• Dissociation between prefrontal cortical and hippocampal contributions to performance of the TUNL task. • Prefrontal cortex lesions result in delay-dependent, but not separation-dependent impairments on TUNL. • Prefrontal cortex lesions result in modest impairments under high interference conditions of TUNL.

## 1 Introduction

The neural structures that support the retention of memories over time has been a subject of intense research in cognitive neuroscience. Recently, however, researchers have become increasingly interested in the putative process of *pattern separation*, through which memories are stored as unique representations that are resistant to confusion (Clelland et al., 2009; Gilbert, Kesner, & DeCoteau, 1998; Yassa & Stark, 2011). It remains unclear, however, to what extent these two processes can be neurally dissociated. To achieve this aim, both memory across a delay and pattern separation must be assessed using the same procedure. There is some very intriguing evidence using such an approach. Kesner and colleagues, for example, provided evidence that hippocampus lesions can impair both memory across a delay, and pattern separation (Gilbert et al., 1998; Kesner, Lee, & Gilbert, 2004; Lee & Kesner, 2002, 2003). However more selective dentate gyrus lesions could impair memory in a separation-dependent manner (Gilbert, Kesner, & Lee, 2001); CA3 lesions, in contrast, did not produce the same separation-dependent deficit, but rather impaired memory at all separations, and at the shortest delays (Gilbert & Kesner, 2006).

Subsequent studies using a number of different approaches have provided further evidence that spatial pattern separation involves cells within the dentate gyrus (Clelland et al., 2009; Goodrich-Hunsaker, Hunsaker, & Kesner, 2008; Kesner, 2007; McHugh et al., 2007). In contrast, the structure most often associated with the process of working memory – “holding information on-line” across a delay interval – is the prefrontal cortex (PFC) (Brown & Bowman, 2002; Courtney, Petit, Haxby, & Ungerleider, 1998). In the present study, we tested the hypothesis that whereas the hippocampus is important for both memory across a short delay and pattern separation (Kesner et al., 2004; Talpos et al., 2010), the PFC is likely to be important only for the former, with spatial pattern separation the selective domain of the dentate gyrus. To test this idea, we used the trial-unique delayed nonmatching-to-location (TUNL) task, developed to assess spatial working memory and pattern separation function using trial-unique locations on a touch-sensitive screen (Talpos et al., 2010). These authors examined task performance after excitotoxic lesions of the hippocampus on performance of TUNL under conditions in which either delay, or spatial separation, was varied parametrically. Hippocampal lesions had no effect at minimal delays and short separations, but significantly impaired performance when the delay period was increased, *or* the separation decreased. In the present study the effects of medial prefrontal cortex (mPFC) lesions were examined under these same conditions. We also tested the additional hypothesis that mPFC lesions would increase susceptibility to interference (Badre & Wagner, 2005; Jonides & Nee, 2006; Postle, Brush, & Nick, 2004).

## 2 General materials and methods

### 2.1 Subjects

Male 250–275 g Lister Hooded rats were obtained from Harlan, UK. Rats were group housed on a reverse light–dark schedule (lights on 7 pm–7 am) and tested during the dark phase. A restricted diet was employed to maintain rats at no less than 85% of free-feeding weight, with water available ad libitum. Rats were habituated to the facility and handling for one week prior to any behavioural training. This experiment was conducted in accordance with the United Kingdom Animals (Scientific Procedures) Act, 1986.

### 2.2 Apparatus

Med Associates (Vermont, USA) rat chambers were similar to those used in previous touchscreen studies (Talpos et al., 2010). The inner chamber is 30 cm wide, 25 cm deep, and 25 cm high consisting of a metal frame with clear Perspex walls. The floor consisted of stainless steel bars spaced 1 cm apart and 3 cm above a tray lined with filter paper. The touchscreen monitors register touch by infrared detectors (Craft Data Ltd., Bucks, UK; ELO Touchsystems, Wiltshire, UK; Displaze, Aylesbury, UK) interfaced by ELO touchscreen software (ELO Touchsystems Inc). The touchscreen monitor (4 cm × 29 cm viewable area, Craft Data Ltd., Chesham, UK) was covered by a black Perspex mask to create 14 active response windows 2 cm by 2 cm, separated by 0.9 cm and raised 16.5 cm from the floor. A spring-hinged ‘shelf’ was attached 16 cm above the grid floor. This shelf was at a 90° angle to the mask and had a depth of 6 cm with a width of 20.5 cm. Masks were attached to the screen leaving a gap of 5 mm between the mask and monitor to ensure that it would not trigger the touchscreen area. On the wall opposite from the monitor was a food magazine (ENV-200R2M) equipped with a 3 W light and infrared detector to register nose pokes (Med Associates Inc., Vermont, USA). The magazine was serviced by a pellet dispenser (Med Assoc. ENV-203-45) delivering 45 mg 5-TUL AIN-76A dustless pellets (TestDiet, Indiana, USA). Above the food magazine was a house light (3 W, Med Assoc. ENV-215M), and tone generator (Med Assoc. ENV-223HAM). Each operant box is housed within a sound-attenuating chamber equipped with a 28 V DC fan. The boxes and monitors were controlled using IBM Netvista and Dell Optiplex computers running custom programs written in Microsoft Visual Basic 6.0.

### 2.3 Behavioural methods

**Fig. 1:**
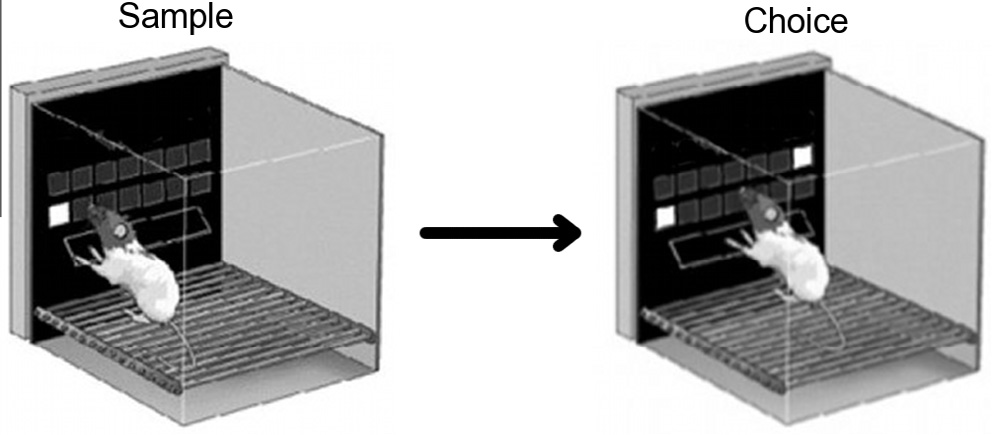
The TUNL task. Images adapted from Talpos et al. (2010). A large separation condition is shown.

The TUNL task consists of two phases: sample and choice. At the sample phase one location within a grid of fourteen squares is illuminated. The rat must respond to the illuminated sample location, then return to the rear food magazine (sample is rewarded in 33% of trials) to initiate the choice phase. During the choice phase the sample square and a novel square are illuminated, and the rat must correctly non-match by selecting the novel square (Fig. 1). A delay can be placed between the sample and choice phases to tax working memory.

As previous discussions of the touchscreen have highlighted, an advantage of this method over two-lever tests such as in delayed non-matching to position (DNMTP) is that stimulus options are not limited to merely two locations (Talpos, Dias, Bussey, & Saksida, 2008). Although the TUNL task is similar to DNMTP in that two locations are used during a given trial, any of the thirteen alternative locations can serve as the correct stimulus on choice. Thus, unlike in DNMTP, the animal cannot know, during the delay, which location will be correct on choice – and therefore cannot orient toward it. Indeed, systematic video analysis of TUNL showed little evidence for performance-enhancing mediating behaviours (Talpos et al., 2010). The use of multiple locations confers an additional advantage; namely, pairs of locations can be chosen which are either close together, far apart, or somewhere in between. This allows assessment of pattern separation alongside the assessment of memory across a delay.

Pretraining followed a similar procedure to that previously reported (Talpos et al., 2010). Session length was 80 trials (sample + choice) or 1 h, with a 20 s intertrial interval (ITI) and no programmed delay between sample and choice phase. The trial structure of TUNL is described in Table 1.

Rats were trained to stable above-chance performance across separations and the 14 best performers were taken forward to the task proper. Rats were baselined pre-surgery on three conditions presented in separate sessions (two blocks of three sessions per condition): large separation, no (that is, minimum possible) delay (LND); large separation with 6-s delay (LWD); and small separation, no delay (SND). Large separation was defined as five locations as horizontal distance between active choice locations. Small separation was defined as two locations as horizontal distance between active choice locations. Each condition was given as a session of 40 trials in 1 h, cycling through the three conditions across days and punctuated with sessions of all separations and no programmed delay (as during acquisition) to minimise the adoption of possible mediating behaviours. Rats were assigned to sham and lesion groups based on baseline performance. Post surgery testing was conducted similarly to pre-surgery baselining. Initial short sessions of 10 and 20 sessions were used to ensure all animals were ready to complete sessions, before cycling though full sessions of all three conditions.

After exploration of LND, LWD, and SND, an interference condition was also tested to investigate whether early trials could interfere with later trials in a given session. In spatial working memory tasks massed presentation of trials (increasing the number of trials and decreasing the interval between trials) has been shown to result in proactive interference (Cohen, Reid, & Chew, 1994; Hoffman & Maki, 1986). Thus, in the interference condition the sessions were 60 trials or 1 h long, large separation during choice only, but without ITI or delay.

### 2.4 Surgery

The mPFC lesions generally followed the protocol established by Birrell and Brown (2000). Lesions were centred on prelimbic and infralimbic cortex. Rats were anaesthetized with 5% isoflurane and maintained at 2% during surgery (IsoFlo isoflurane, Abbott Labs, UK administered via VetTech Solutions Ltd. apparatus, UK). They were positioned in a stereotaxic frame (David Kopf Instruments) fitted with atraumatic ear bars (Kopf 955) with nose bar set to +5 mm. Bilateral injections (four in total) of 0.2 μL of 0.06 M ibotenic acid or vehicle were made at AP + 3.5 mm; L ± 0.6 mm; V − 5.2 mm and AP + 2.5 mm; L ± 0.6 mm; V − 5 mm relative to skull surface bregma using a custom infusing line connected to a 10 μL Hamilton syringe and Harvard Instruments (Holliston, Massachusetts, USA) ‘Pump 11’ infusion pump. One sham animal was culled perioperatively due to a poor reaction to the anaesthesia. Subjects recovered for at least one week with ad libitum food and water prior to behavioural testing.

### 2.5 Histology

Rats were terminally anaesthetized with sodium pentobarbitone (Dolethal, Vetoquinol, UK) and perfused transcardially with 0.01 M PBS followed by formaldehyde solution (4% paraformaldehyde in PBS). Brains were removed and post-fixed in formaldehyde solution. Prior to sectioning on a freezing microtome brains were transferred into 20% sucrose in 0.01 M PBS and left overnight. Coronal sections (60 μm) were stained with NeuN and lesion locations were mapped onto standardised sections of the rat brain (Paxinos & Watson, 2007).

**Fig. 2:**
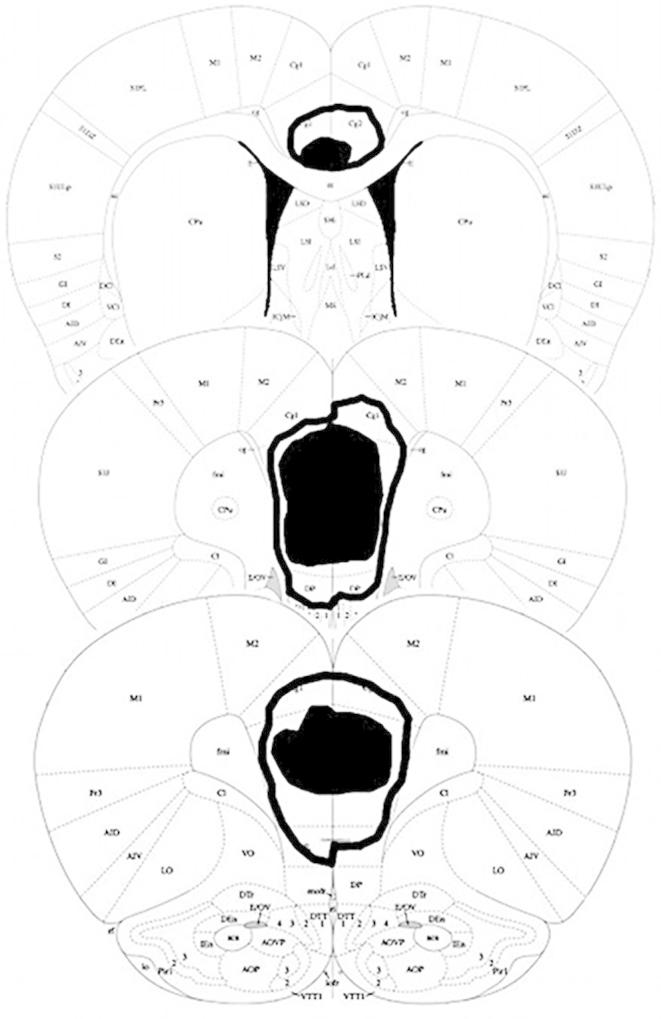
mPFC lesions in the present study. Damage common to all subjects is shown in black. The maximum extent of any damage is shown by the black line. Coronal sections are taken at 3.72 mm, 2.76 mm, and 1.08 mm anterior to bregma. Images adapted from Paxinos and Watson (2007).

**Fig. 3:**
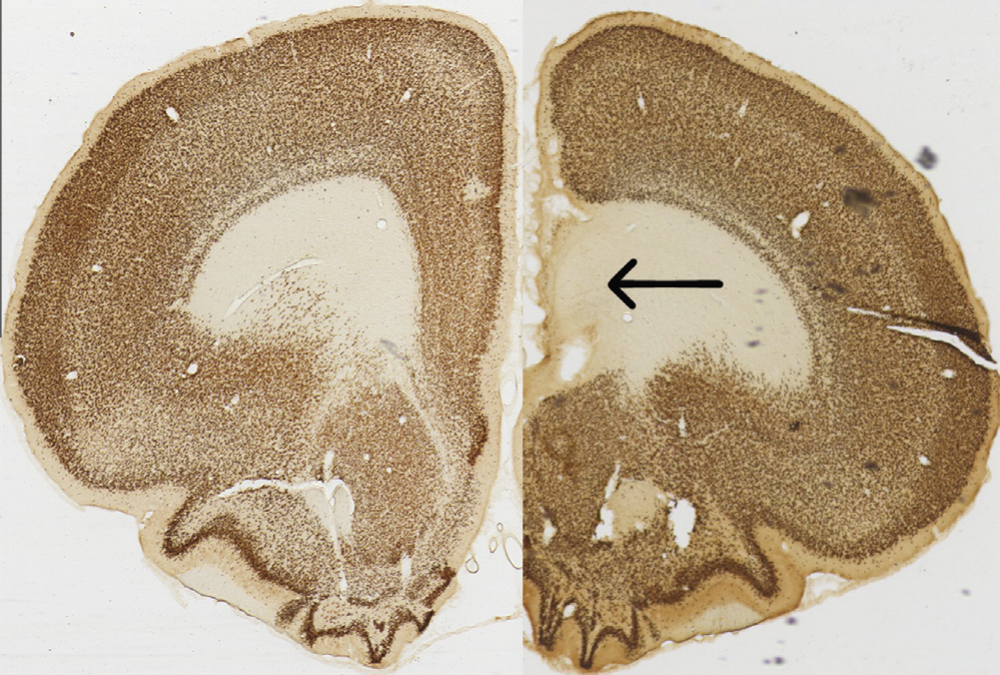
Composite image of sham on left and lesion on right.

Lesions were centred on prelimbic cortex, with damage extending into infralimbic cortex and overlying anterior cingulate (Figs. 2 and 3). One lesioned animal was excluded from data analysis due to an incomplete lesion.

## 3 Results

**Fig. 4:**
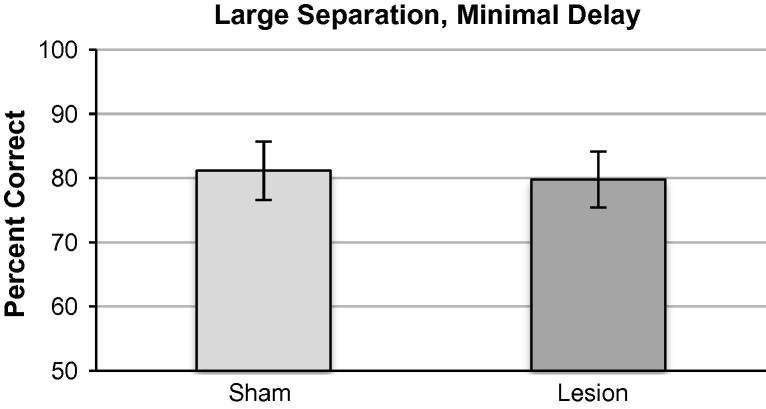
Large separation, minimal delay condition (LND: a horizontal distance of five locations between active choice locations). There was no effect of lesion on accuracy in this condition. Data presented as means ± 1SEM.

**Fig. 5:**
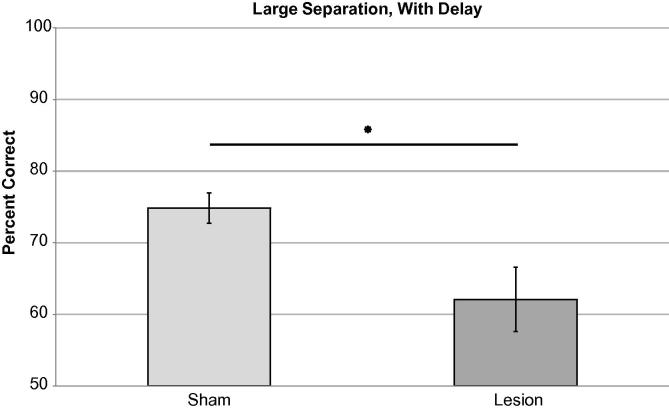
Large separation with delay condition (LND: a horizontal distance of five locations between active choice locations and a 6-s programmed delay). There was a significant effect of lesion on accuracy in this condition. Data presented as means ± 1SEM. ^∗^ = *p* < 0.05.

There was no significant effect of lesion in the LND condition (Fig. 4). Performance was stable across three blocks of testing (three sessions/block repeated measures ANOVA *F*_(1,8) _= 2.44, *p* = 0.22) with no effect of lesion on percent correct *F*_(1,8) _= 0.01, *p* = 0.93 and no lesion × session interaction *F*_(1,8) _= 0.29, *p* = 0.75).mPFC impaired accuracy in the LWD condition (Fig. 5). Performance was stable across three blocks of testing (three sessions/block repeated measures ANOVA *F*_(2,14) _= 2.77, *p* = 0.097) with a significant effect of lesion on overall mean percent correct (*F*_(1,8) _= 6.71, *p* = 0.021) and no lesion x session interaction (*F*_(2,14) _= 1.75, *p* = 0.22).

**Fig. 6:**
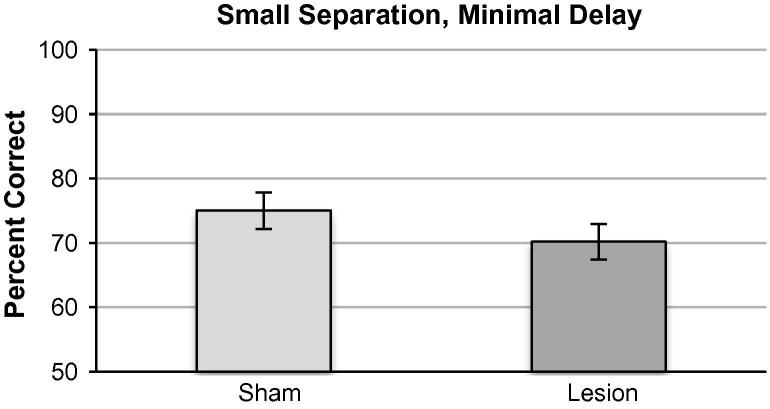
Small separation minimal delay condition (SND: a horizontal distance of two locations between active choice locations). There was no effect of lesion on accuracy in this condition. Data presented as means ± 1SEM.

No significant difference was seen in performance between sham and lesion in the SND condition (Fig. 6). Performance was stable across three blocks of testing (three sessions/block repeated measures ANOVA *F*_(2,14) _= 1.11, *p* = 0.36) with no significant effect of lesion on mean percent correct *F*_(1,8) _= 1.55, *p* = 0.25) or lesion × session interaction (*F*_(2,14) _= 0.086, *p* = 0.92).

**Fig. 7:**
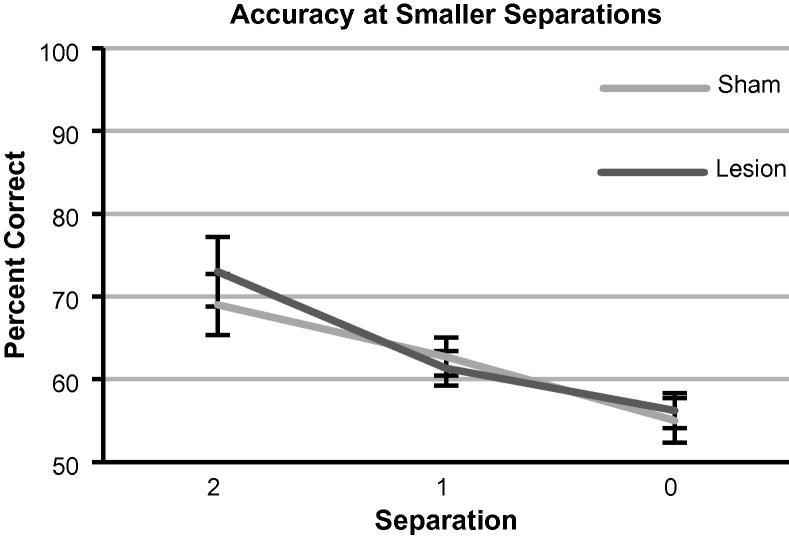
Accuracy at smaller separations. As both groups completed the SND condition at a relatively high level of accuracy, a separation challenge was conducted where one block was given of each smaller separation (sessions cycled through separations, punctuated with sessions of all separations). While smaller separations were more difficult, there was no difference between sham and lesion. Separation shows the number of horizontal locations in between the active choice locations (0 being adjacent). Data presented as block means ± 1SEM.

These results suggested that mPFC lesions have no effect on pattern separation ability. To test this idea stringently, rats were tested on still smaller separations. Decreasing separation led to increasing difficulty, but equally so for both groups (Fig. 7). Repeated measures ANOVA showed a significant effect of separation on accuracy *F*_(2,20) _= 92.5, *p* = <0.001), but no main effect of lesion *F*_(1,10) _= 0.54, *p* = 0.48) or lesion × separation interaction *F*_(2,20) _= 1.03, *p* = 0.37). While performance decreased significantly with smaller separations, accuracy was still greater than chance when choice locations were adjacent (sham *t*_(6) _= 2.5, *p* = 0.046; lesion *t*_(5)_=2.95, *p* = 0.032).

**Fig. 8:**
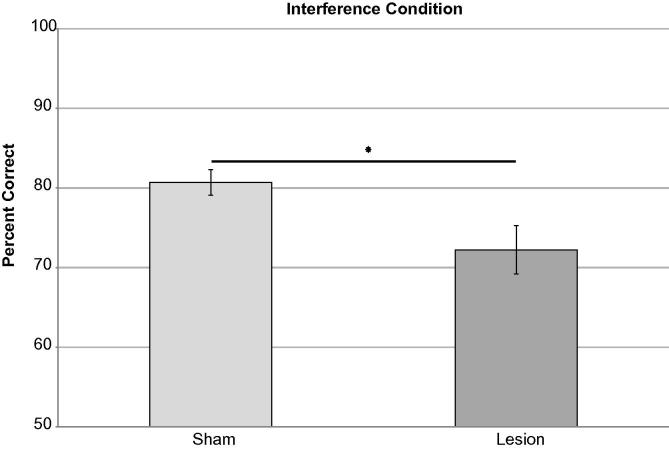
Interference condition. There was a significant effect of lesion on accuracy in this condition. Data presented as means ± 1SEM. ^∗^ = *p* < 0.05.

Interference was then increased by removing the inter-trial interval (ITI), while maintaining minimal delay. An overall impairment was observed in this high interference condition, with lesioned animals significantly less accurate than shams (Fig. 8). This impairment was obtained in the minimal delay condition under which mPFC were significantly impaired, when a 20s ITI was used. Performance was stable across three blocks of testing (three sessions/block repeated measures ANOVA *F*_(2,12) _= 1.77, *p* = 0.21) with a significant effect of lesion on overall mean percent correct (*F*_(1,8) _= 7.92, *p* = 0.043) and no lesion × session interaction (*F*_(2,12) _= 1.64, *p* = 0.55). There was no significant difference in performance early versus late in the session (paired *t*-tests sham *t* = 0.038, *p* = 0.73; lesion *t* = 0.027, *p* = 0.80).

There was no effect of lesion on reaction times or magazine latency in any condition (Tables 2–4).

## 4 Discussion

The major finding of the present study was that prefrontal cortex lesions impaired memory across a delay in TUNL, while completely sparing spatial pattern separation. In addition, mPFC lesions impaired performance under conditions of increased interference (no ITI) under minimal delay conditions, but not when a long (20s) ITI was used. These data are consistent with previous investigations of prefrontal cortex function and working memory in humans and non-human primates (Castner, Goldman-Rakic, & Williams, 2004) and similar lesions in rats (Delatour & Gisquet-Verrier, 1996; Sloan, Good, & Dunnett, 2006), and with the suggestion that PFC lesions can increase susceptibility to interference in memory (Granon, Vidal, Thinus-Blanc, Changeux, & Poucet, 1994; Ragozzino & Rozman, 2007). In the present study, PFC lesions led to impairments at a long, but not a short delay. Although others have found delay-dependent impairments after lesions of the PFC (Sloan et al., 2006b), Others report delay-*in*dependent impairments (Porter et al., 2000; Chudasama and Muir (1997); for review see Dudchenko, Talpos, Young, and Baxter (2012). One reason for this discrepancy could be differences between the lesions in these various studies. However, another salient difference is the testing method; locations in TUNL are relatively trial-unique compared to DNMTP in a 2-lever operant chamber. Indeed, the repetition of the same two locations across all trials in DNMTP likely increases interference, in some cases making the task more sensitive to PFC damage, consistent with the increased susceptibility to interference demonstrated in the present study. Thus TUNL may more readily enable detection of delay-dependent impairment following at least some experimental manipulations.

**Fig. 9:**
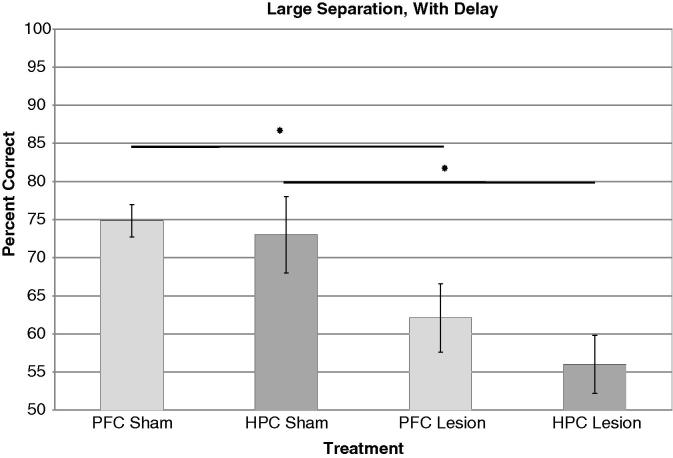
PFC and HPC lesions on LWD condition. HPC lesion data adapted from Talpos et al. (2010).

Particularly striking were the clear contrasts – but also the similarities – between the pattern of impairment following mPFC lesions and that following hippocampal dysfunction. Both prefrontal cortex lesions and hippocampal lesions produced significant impairments in the presence of a 6 s delay (Fig. 9). However, whereas lesions of the hippocampus impaired pattern separation (as shown by impairments at small separations and not large ones), mPFC lesions in the present study had no effect on pattern separation at minimal delay, even when separations were made so small as to bring performance well down from ceiling. This pattern suggest that whereas both the hippocampus and mPFC are essential for retention of memories across a short delay, and likely functionally interact in this regard, only the hippocampus is necessary for spatial pattern separation. In addition, the finding of no impairment in difficult pattern separation conditions shows that PFC lesions do not simply produce impairments in any condition in which the task is made more difficult.

Other studies have reported similar dissociations between PFC and hippocampus lesions on tests of memory. For example, inactivation of the mPFC (prelimbic and infralimbic) or dorsal hippocampus on a spatial delayed-alternation task showed that while both manipulations resulted in delay-dependent impairments in accuracy, mPFC lesions did not impair reference memory or choice latency, which was impaired following hippocampal inactivation (Yoon, Okada, Jung, & Kim, 2008). To investigate the nature of the prefrontal–hippocampal interactions that underlie working memory, Wang and Cai (2006) investigated the effects of unilateral or bilateral mPFC inactivation (prelimbic), unilateral or bilateral hippocampus inactivation (ventral), or a crossed ‘disconnection’ preparation involving unilateral inactivation of the hippocampus along with contralateral inactivation of mPFC. Behavioural probes on a delayed spatial alternation task demonstrated that unilateral inactivation of either structure did not impair performance, bilateral inactivation of either structure impaired performance, and the unilateral-contralateral inactivation impaired performance. Earlier Seamans, Floresco, and Phillips (1998) had used a more subtle disconnection manipulation of PFC-hippocampal circuitry, showing that unilateral injection of a dopamine D1 antagonist into mPFC combined with contralateral injection of lidocaine into the hippocampus impaired memory in a radial arm maze. These studies provide compelling evidence for the putative interaction of mPFC and hippocampus in the service of memory.

Such prefrontal cortex-hippocampus interaction may be of particular relevance to schizophrenia, as it has been widely acknowledged that structural and functional changes in both the hippocampus and prefrontal cortex play an important role in the pathophysiology of the disorder. Furthermore, schizophrenic brains show altered connectivity between hippocampus and prefrontal cortex (Meyer-Lindenberg et al., 2005), and impaired hippocampal-prefrontal synchrony during performance on a working memory task has been observed in a mouse with a microdeletion on human chromosome 22 (22q11.2), a genetic risk factor for schizophrenia (Sigurdsson, Stark, Karayiorgou, Gogos, & Gordon, 2010). This finding mirrors human data where abnormal coupling is seen between the prefrontal cortex and hippocampus in patients with schizophrenia and healthy carriers of *rs1344706* risk genotypes (Esslinger et al., 2009). Thus the TUNL task, with its ability to assess working memory and pattern separation within the same task, and indeed demonstrate dissociations between the two, may be a particularly useful paradigm for studying cognition in rodent models of schizophrenia.

Dissociation in the pattern separation condition is also consistent with previous literature. A number of studies have linked the hippocampus, and dentate gyrus in particular, with pattern separation (Clelland et al., 2009; Creer, Romberg, Saksida, van Praag, & Bussey, 2010; Gilbert et al., 1998, 2001; Yassa & Stark, 2011). The present TUNL data are supported by a similar touchscreen study that demonstrated that lesions of the dorsal hippocampus impaired spatial discrimination when the locations were close together, but not far apart (McTighe, Mar, Romberg, Bussey, & Saksida, 2009), indicating an impairment in pattern separation. However to our knowledge this is the first time PFC lesions have been assessed on pattern separation.

mPFC lesions in the present study also increased susceptibility to interference. Development and investigation of the interference condition was originally inspired by theories of proactive interference: the memory of earlier events interfering with memory of more recent events (Postman & Underwood, 1973). In a radial arm maze proactive interference has been described as ‘intertrial’ when resulting from a previous trial, or ‘intratrial’ when resulting from visiting other arms within a trial (Cohen, Sturdy, & Hicks, 1996). Intertrial proactive interference has been observed in both radial arm and alternation tasks when trials are massed (Dale & Roberts, 1986; Grant, 1981; Roberts & Dale, 1981). In particular, lowering the ITI decreased performance, which was ameliorated with a longer ITI (Cohen et al., 1994). These intertrial effects have been described as resulting from a failure in temporal discrimination: in the radial arm maze this manifests as confusion over whether arms to be visited in later choices were visited earlier in the current trial, or in previous trials (Roberts & Dale, 1981). Intratrial interference has been demonstrated in the T-maze delayed alternation task by adding forced pre-study runs (Gordon & Feldman, 1978; Gordon & Schlesinger, 1976; Grant, 1980). An analogous task in the radial arm maze showed impaired performance following forced visits to arms in a pre-study phase (Hoffman & Maki, 1986), with similar effects in a subsequent study (Cohen et al., 1996).

Human studies of working memory function have demonstrated differences in item-specific and item-nonspecific proactive interference (PI). Item-specific proactive interference occurs when a negative probe matches an exemplar from a previous trial (Monsell, 1978). Item-specific PI has been linked to longer reaction times and the memory probe/response epoch in Brodmann’s area 45 of the left inferior prefrontal cortex (Jonides, Badre, Curtis, Thompson-Schill, & Smith, 2002; Nelson, Reuter-Lorenz, Sylvester, Jonides, & Smith, 2003; Postle et al., 2004). Item-nonspecific PI results from the accumulation of irrelevant memory from previous trials—and thus related to processes of forgetting in short-term and working memory (Bunting, 2006; May, Hasher, & Kane, 1999; Wickens, Born, & Allen, 1963). Studies to examine the neural basis of item-nonspecific PI suggest that similar mechanisms underlie both types of interference. Postle et al. (2004) found that item-nonspecific effects were also linked to probe epochs in left Brodmann’s area 45. Further studies have supported a key role of the left inferior frontal gyrus in proactive interference ((Badre & Wagner, 2005; Mecklinger, Weber, Gunter, & Engle, 2003) reviewed in Badre and Wagner (2007) and Jonides and Nee (2006)).

Results from the present study are consistent with these findings. However it is worth noting that while mPFC did result in impaired performance of the interference condition, there was no difference in accuracy early versus late in the session, indicating that there was not a significant cumulative effect of early trials interfering with later trials. This finding does not, however, discount an immediate effect of intertrial interference, as the immediately preceding trials could interfere with the current choice and result in consistently poorer performance. It is also worth noting that another way the lack of an ITI may have resulted in an increased cognitive demand, leading to increased dependence on PFC, may have been the lack of an obvious ITI to cue whether the phase of the trial was a sample or a choice. The resulting ambiguity could conceivably render the task more susceptible to dysfunction of the mPFC. However it is worth noting that performance of control animals in the minimal delay conditions with and without ITI were very similar; thus the no ITI condition does not appear to be substantially more difficult for normal animals. Our final caveat regarding our findings is that the sample sizes were relatively small, resulting is relatively low power and effects that were, although significant, not highly so. We would recommend that future studies using this paradigm use higher group numbers to ensure enough power to detect differences, especially when using more subtle perturbations of the system than the lesion approach used here.

## 5 Conclusion

Combined with the results of previous work by Talpos et al. (2010), the current study demonstrates both similar, and different contributions of prefrontal cortex and the hippocampus to working memory and pattern separation. While prefrontal cortex is necessary when working memory load is increased, it is not necessary even for the most difficult pattern separation conditions. These findings also demonstrate the utility of the TUNL paradigm for research into the functions of prefrontal-hippocampal circuitry, and rodent models of disorders of cognition such as Alzheimer’s disease (Braak & Braak, 1997; Dickerson & Eichenbaum, 2010; Vargha-Khadem et al., 1997). Furthermore as PFC-hippocampal interaction is abnormal in schizophrenia, and both working memory and pattern separation impairments are linked to schizophrenia (reviewed in Goldman-Rakic (1999), Kuperberg and Heckers (2000), and Tamminga, Stan, and Wagner (2010)), the TUNL task may prove to be a particularly valuable tool for preclinical research into schizophrenia.

## Figures and Tables

**Table 1:** Trial structure of TUNL.

House light and magazine light on	
Nose-poke to magazine → Magazine light off, sample location illuminated	
Nose-poke to sample location →	33.3% of trials: tone, magazine light 1s, reward
	All trials: start delay timer
Delay timer end → Magazine light illuminated	
Nose-poke to magazine → Choice phase locations illuminated	
Incorrect response → House light off for 5 s followed by correction trial	
Correct response → Tone, magazine light on, reward delivered	
Reward collected → Magazine light off, ITI begins	

**Table 2:** Mean sample reaction time across conditions ± 1SEM. Sample reaction time is the duration between initiating a trial and responding to the sample location. Reaction times were log transformed prior to analysis. No significant difference was seen between sham and lesion in any condition.

Condition	Mean sample reaction time			
	Sham	Lesion	Significance	
LND	3.36 ± 0.21	3.84 ± 0.89	*t* = 0.58	*p* = 0.82
LWD	3.32 ± 0.44	3.32 ± 0.13	*t* = 0.20	*p* = 0.85
SND	3.16 ± 0.61	3.69 ± 0.24	*t* = 0.86	*p* = 0.42
Interference	4.23 ± 1.22	6.4 ± 2.38	*t* = 0.97	*p* = 0.37

**Table 3:** Mean choice reaction time across conditions ± 1SEM. Choice reaction time is the time taken to select a location during the choice phase. Reaction times were log transformed prior to analysis. No significant difference was seen between sham and lesion in any condition.

Condition	Mean choice reaction time			
	Sham	Lesion	Significance	
LND	4.01 ± 0.46	3.50 ± 0.23	*t* = 0.74	*p* = 0.48
LWD	3.37 ± 0.56	3.40 ± 0.45	*t* = 0.20	*p* = 0.85
SND	4.37 ± 0.30	4.15 ± 0.12	*t* = 0.69	*p* = 0.52
Interference	3.87 ± 0.82	4.68 ± 0.29	*t* = 0.44	*p* = 0.67

**Table 4:** Mean magazine latency across conditions ± 1SEM. Magazine latency is the duration between selecting a choice location and collecting the reward. Latencies were log transformed prior to analysis. No significant difference was seen between sham and lesion in any condition.

Condition	Mean magazine latency			
	Sham	Lesion	Significance	
LND	1.27 ± 0.12	1.34 ± 0.15	*t* = 0.47	*p* = 0.66
LWD	1.34 ± 0.18	1.07 ± 0.07	*t* = 1.08	*p* = 0.32
SND	1.07 ± 0.12	1.11 ± 0.06	*t* = 0.54	*p* = 0.61
Interference	1.18 ± 0.17	1.10 ± 0.06	*t* = 0.22	*p* = 0.83
